# Lingual Denervation Improves the Efficacy of Anti-PD-1 Immunotherapy in Oral Squamous Cell Carcinomas by Downregulating TGFβ Signaling

**DOI:** 10.1158/2767-9764.CRC-23-0192

**Published:** 2024-02-15

**Authors:** Zhuo-Ying Tao, Leilei Wang, Wang-Yong Zhu, Gao Zhang, Yu-Xiong Su

**Affiliations:** 1 Division of Oral and Maxillofacial Surgery, Faculty of Dentistry, The University of Hong Kong, Hong Kong.; 2 Division of Applied Oral Sciences and Community Dental Care, Faculty of Dentistry, The University of Hong Kong, Hong Kong.

## Abstract

**Purpose:**

Intratumoral nerve infiltration relates to tumor progression and poor survival in oral squamous cell carcinoma (OSCC). How neural involvement regulates antitumor immunity has not been well characterized. This study aims to investigate molecular mechanisms of regulating tumor aggressiveness and impairing antitumor immunity by nerve-derived factors.

**Experimental Design:**

We performed the surgical lingual denervation in an immunocompetent mouse OSCC model to investigate its effect on tumor growth and the efficacy of anti-PD-1 immunotherapy. A trigeminal ganglion neuron and OSCC cell coculture system was established to investigate the proliferation, migration, and invasion of tumor cells and the PD-L1 expression. Both the neuron-tumor cell coculture *in vitro* model and the OSCC animal model were explored.

**Results:**

Lingual denervation slowed down tumor growth and improved the efficacy of anti-PD-1 treatment in the OSCC model. Coculturing with neurons not only enhanced the proliferation, migration, and invasion but also upregulated TGFβ-SMAD2 signaling and PD-L1 expression of tumor cells. Treatment with the TGFβ signaling inhibitor galunisertib reversed nerve-derived tumor aggressiveness and downregulated PD-L1 on tumor cells. Similarly, lingual denervation *in vivo* decreased TGFβ and PD-L1 expression and increased CD8^+^ T-cell infiltration and the expression of IFNγ and TNFα within tumor.

**Conclusions:**

Neural involvement enhanced tumor aggressiveness through upregulating TGFβ signaling and PD-L1 expression in OSCC, while denervation of OSCC inhibited tumor growth, downregulated TGFβ signaling, enhanced activities of CD8^+^ T cells, and improved the efficacy of anti-PD-1 immunotherapy. This study will encourage further research focusing on denervation as a potential adjuvant therapeutic approach in OSCC.

**Significance:**

This study revealed the specific mechanisms for nerve-derived cancer progression and impaired antitumor immunity in OSCC, providing a novel insight into the cancer-neuron-immune network as well as pointing the way for new strategies targeting nerve-cancer cross-talk as a potential adjuvant therapeutic approach for OSCC.

## Introduction

According to the Global Cancer Statistics 2020, oral cancer accounts for 2% of all new cases and 1.8% of all deaths from cancer (1). Oral squamous cell carcinoma (OSCC), occupying the highest proportion of oral cancer, has poor prognosis, and even in developed countries such as the United States, the 5‐year survival rate is only 65% (2). Much effort has been exerted to improve the prognosis of oral cancer, but little progress has been made in recent decades. Accumulative evidence contributes to the relationship between nerve-tumor cross-talk and poor clinical outcomes in cancer (3). Preclinical research has confirmed that human peripheral nerves significantly promote the aggressiveness of tumor cells from patients with OSCC (4). Clinically, it has been long recognized that the nerve density within the tumor is strongly associated with local recurrence, cervical lymph node metastasis, and lower survival in patients with OSCC (5, 6). Moreover, overexpression of the β2-adrenergic receptor (a neurotransmitter receptor for noradrenaline) correlates with a more advanced clinical stage and preoperative lymphatic metastasis in OSCC clinically (7). The specific mechanism has been revealed by preclinical research from *Nature* that oral cancer cells with deficient or mutant TP53 lead to an adrenergic transdifferentiation of innervated lingual nerves, which aids tumor growth (8). Besides neurotransmitters, other neuroactive molecules including neuropeptides, neurotrophins released by intratumoral nerve fibers also regulate cancer invasion and metastasis (9).

Checkpoint blockade immunotherapy, centered on blocking immune inhibitory receptors including programmed cell death 1 (PD-1), programmed cell death-ligand 1 (PD-L1), and CTL-associated protein 4 (CTLA4), benefits no more than one-fifth of patients, possibly due to inadequate immune activation (10). The unresponsiveness to immunotherapy represents a big and emerging challenge in clinical practice. However, few studies focus on the relationship between neural involvement and antitumor immunity in cancer. A recent study has demonstrated that the high expression of nerve growth factor receptors on tumor cells predicts anti-PD-1 therapy resistance in patients with melanoma (11), which provides evidence for the correlation between neural signaling and immunotherapy responses. Interestingly, our previous bioinformatic analysis on head and neck cancer transcriptome data from The Cancer Genome Atlas revealed that the neural-related differential expressed genes (DEG) had a predictive role in both the survival and the benefit of immunotherapy in head and neck squamous cell carcinoma (HNSCC; 12). Issues awaiting clarification include the underlying mechanisms for the nerve-tumor cross-talk leading to tumor progression and immunotherapy resistance in OSCC.

Tumor-derived TGFβ is a multifunctional cytokine within the tumor microenvironments, and TGFβ signaling is central to both tumorigenesis and immune evasion (13). TGFβ can facilitate tumor progression by altering the metabolic reprogramming process of cancer-associated fibroblasts (14), promoting epithelial-to-mesenchymal transitions (15) and angiogenesis (16). Multiple studies have revealed its roles in tumor immune evasion and poor responses to cancer immunotherapy, as evidenced by the fact that most immune cells respond to TGFβ strongly and transform to immunosuppressive phenotypes (17–19). Whether TGFβ signaling participates in the nerve-derived tumor progression or immunotherapy resistance is unclear. Therefore, this study aimed to investigate the role of intratumoral nerves in both the tumor progression and checkpoint blockade immunotherapy in OSCC, as well as explore the impact of TGFβ signaling in this cancer-neuro-immune communication, to provide evidence for targeting nerve-cancer cross-talk as a potential adjuvant therapeutic approach for OSCC control.

## Materials and Methods

### *In Vivo* Mice Tumor Models

C57BL/6 mice (female, 6–8 weeks old) were purchased from the center for comparative medicine research of the University of Hong Kong (Hong Kong). All animal research procedures were approved by the committee on the use of live animals in teaching and research (CULATR, 5806-21) of the University of Hong Kong (Hong Kong). The syngeneic orthotopic oral cancer model was established by subcutaneous inoculation of Murine oral cancer 1 (MOC1, RRID: CVCL_ZD32) tumor cells (30,000 cells in 30 µL) to the dorsum of tongue in C57BL/6 mice, and surgical lingual denervation (LD) was performed one week before or after cancer cell inoculation. LD was performed as described previously (20). Mice were anesthetized and prepared, and a skin incision below the lower border of the mandible was performed to expose the bilateral lingual nerves in the neck, which were transected between the anterior belly of the digastric and masseter muscles. Although the proximal and distal stumps of the transected lingual nerve were separated, a 5-mm section was resected of each stump to minimize regeneration. For sham LD surgery, bilateral lingual nerves were exposed without sections. Mice were injected intraperitoneally with anti-PD-1 (αPD-1, Bio × Cell, BE0146, 200 µg/mouse in 0.2 mL InVivoPure pH 7.0 Dilution Buffer) or IgG2a (Bio × Cell, BE0089, 200 µg/mouse in 0.2 mL InVivoPure pH 6.5 Dilution Buffer) every 3 days for four times in the oral cancer model. One week after the last αPD-1 or IgG treatment, the mice were sacrificed and the tongue tissues were collected. The tumor size was measured and tumor volume = 0.5 × length (mm) × width (mm)^2^.

### Cell Line and Cell Culture

Human tongue squamous cell carcinoma cell line SCC-15 was purchased from the ATCC (CRL-1623, RRID: CVCL_1681), and cultured in complete DMEM/F12 medium (DMEM: Nutrient Mixture F-12, Gibco, Thermo Fisher Scientific, 11320082) supplied with 40 µg/L hydrocortisone (Sigma-Aldrich, H0888), 10% FBS, and 1% penicillin/streptomycin at 37°C in a humidified atmosphere with 5% CO_2_. The MOC1 cell line was provided by Dr. R. Uppaluri (Washington University School of Medicine, St. Louis, MO). MOC1 cells were cultured in IMDM (Iscove's Modified Dulbecco's Medium) MOC line media, which was a 2:1 mixture of IMDM (Gibco, Thermo Fisher Scientific, 12440053) and F12 medium (Gibco, Thermo Fisher Scientific, 11765054) supplied with 5 mg/L insulin, 40 µg/L hydrocortisone, 5 µg/L EGF, 10% FBS, and 1% penicillin/streptomycin. All cell lines were authenticated and not cultured for longer than 15 passages. *Mycoplasma* was screened by a PCR-based detection kit (Beyotime, C0303) every 2 weeks. Primary sensory neurons were isolated from trigeminal ganglion (TG) dissected from 6 to 8 weeks old C57BL/6 mice as described previously (21). The dissected TGs were digested enzymatically with papain (40 U/mL, Worthington, 3126) for 20 minutes at 37°C followed by another 20 minutes of digestion with collagenase II (4 mg/mL, Worthington, 4176)/dispase II (4.6 mg/mL, Sigma-Aldrich, D4693) solution. The myelin and nerve debris of TGs were separated using Percoll (Sigma-Aldrich, P1644) gradient, comprising 12.5% and 28% Percoll in complete L-15 medium (Gibco, Thermo Fisher Scientific, 11415064) supplied with 5% FBS and 1% penicillin/streptomycin. TG primary sensory neurons were plated on laminin-coated coverslips and cultured with complete F12 medium supplied with 5% FBS and 1% penicillin/streptomycin and incubated at 37°C in a humidified atmosphere with 5% CO_2_.

### *In Vitro* Neuron-tumor Cell Coculture Model

Two coculture models of primary TG neurons and oral cancer tumor cells were established. In the neuron-tumor cell coculture system, inserts with 0.4 µm pores (SPL Life Sciences, 35006) were used. In coculture group 1, the lower chamber was seeded with TG sensory neurons for 48 hours, and then tumor cells were seeded in the upper chamber inserts. In coculture group 2, the coculture medium from coculture group 1 was added to the lower chamber and the tumor cells were seeded in the upper insert. The control group was set with tumor cell culture only.

### Cell Viability Assay

Cell viability was evaluated using an 3-[4,5-dimethylthiazol-2-yl]-2,5-diphenyltetrazolium bromide (MTT) Cell Proliferation Assay Kit (Thermo Fisher Scientific, V13154) according to the product instruction. To find the most appropriate coculture time for the neuron-tumor cell coculture model, tumor cells were seeded into 96-well plates (5,000 each well) and treated with the coculture medium of different timepoints (24, 48, and 72 hours) from the coculture group 1 as well as the different complete medium (complete F12 medium and complete DMEM/F12 medium or complete IMDM MOC line media). After incubating at 37°C in a humidified atmosphere of 5% CO_2_ for 24, 48, and 72 hours, the medium of each was removed and 100 µL of fresh culture medium with 10 µL of 12 mmol/L MTT stock solution was added to each well. After labeling the cells with MTT, 85 µL of the medium was removed and 50 µL of DMSO was added to each well. The plate absorbance at 540 nm was set as the cell proliferation index and detected by a SpectraMax M2 microplate reader (Molecular Device) after incubating the plates for 10 minutes at 37°C.

MTT Cell Proliferation Assay was also performed to find the appropriate concentration of galunisertib (LY2157299, MedChemExpress, HY-13226) treatment. A stock solution of 10 mmol/L galunisertib was prepared by dissolving it in 100% DMSO. Serial dilutions of galunisertib (0, 0.01, 0.1, 1, 2.5, 10, and 100 µmol/L) were added to the SCC-15 or MOC1 cell cultures. The cell proliferation index was compared between each group.

### Tumor Cell Migration and Invasion Assay

For migration/invasion assay, 8 µm pores inserts (SPL Life Sciences, 35224) were used to evaluate migration/invasion ability (22). Tumor cells were serum starved overnight before being added to the upper inserts in a serum-free medium. The lower chamber was added with TG sensory neurons in the complete F12 medium or coculture medium from coculture group 1 or complete tumor cell medium as the attractant. For migration assay, tumor cells were allowed to migrate for 24 hours at 37°C. For invasion assays, the upper side of the insert membrane was coated with 30% Matrigel (v/v; Corning, 356234), and tumor cells were allowed to invade for 36 hours. After migration/invasion, cells on the upper side of the membrane were removed, while the migrated/invaded cells on the undersurface of the membrane were fixed in 4% paraformaldehyde for 10 minutes and then stained with 1% crystal violet staining solution for 10 minutes. The images of migrated/invaded cells were captured under a brightfield microscope, and the number of migrated/invaded cells per field view was counted using the cell counter plugins in Image J (NIH, RRID: SCR_003070).

### Wound Healing Assay

The wound healing assay was performed as described previously (23). Tumor cells were seeded into 6-well plates at 95% confluence. After being cultured with serum-free medium for 24 hours, a scratch wound was made using the 200 µL pipette tube at the bottom of the well. Images about the cell migration in each group were captured at 0, 12, 24, and 36 hours at the marked region with the microscope.

### RNA Sequencing and qRT-PCR

Total RNA was extracted from the SCC-15 cells from different groups using a TaKaRa MiniBEST Universal RNA Extraction Kit (TaKaRa, 9767) according to the manufacturer's instructions. The total RNA of different groups was used for Eukaryotic Strand-specific Transcriptome Resequencing performed with the DNBseq platform by BGI [The Sequence Read Archive (SRA): PRJNA1062351]. Gene expression levels were calculated and normalized to transcripts per million mapped reads. DEGs and pathway enrichment analysis were performed by R 4.1.0.

Extracted total RNA was reversed into cDNA using PrimeScript RT reagent Kit (TaKaRa, RR047B). qRT-PCR was performed using the TB Green Premix DimerEraser (TaKaRa, RR091A) in an Applied Biosystems 7500 Real-Time PCR Systems. Gene expression of SCC-15 cells was normalized to β-actin. All the relative expression levels were calculated according to the 2-ΔΔC_t_ method. The primers used for qRT-PCR were as follows: TGFβ1: forward 5′-AACCCACAACGAAAT CTATGAC-3′, reverse 5′-GGAATTGTTGCTGTATTTCTGG-3′; TGFβ2: forward 5′-CTACTTAAT AGCCACTCGTC-3′, reverse 5′-CTAGTCAATGCCCAACAG -3′; TGFβ3: 5′-GGTTTTCCGCTTC AATGTGT-3′, reverse 5′-GCTCGATCCTCTGCTCATTC-3′; PD-L1: forward 5′-TATGGTGGTGCC GACTACAA-3′, reverse 5′-TGCTTGTCCAGATGACTTCG-3′; β-actin: forward 5′-CTAAGTCAT AGTCCGCCTAGAAGCA-3′, reverse 5′-TGGCACCCAGCACAATGAA-3′.

### ELISA

The TGFβ1 level in the supernatants of the coculture groups and the control group were collected and analyzed by ELISA (R&D Systems, DB100C) according to the product instructions. The supernatants from coculture groups were mixed and collected from both upper and lower chambers. The optical density of the plates was read at 540 and 570 nm by the SpectraMax M2 microplate reader (Molecular Device).

### Western Blot Analysis

Tumor cells of different groups were collected for total protein extraction. An equal quantity of proteins was separated by 10% SDS-PAGE and transferred onto polyvinylidene fluoride membranes. After being blocked with 5% skimmed milk in Tris-buffered saline with 0.1% Tween 20 Detergent (TBST) for 2 hours, the membranes were incubated with primary antibodies: TGFβ1 (1:1,000, Abcam, ab215715), PD-L1 (1:1,000, Cell Signaling Technology, 13684), p-SMAD2 (1:500, Cell Signaling Technology, 18338), SMAD2 (1:1,000, Cell Signaling Technology, 5339), and β-actin (1:2,000, Cell Signaling Technology, 4970) at 4°C overnight. Then, the membranes were washed and incubated with anti-rabbit horseradish peroxidase (HRP)-linked secondary antibodies (1:5,000, Abcam, ab6721, RRID: AB_955447) for 2 hours at room temperature, and the blots were visualized using the iBright FL1500 Imaging System (Thermo Fisher Scientific).

### Immunofluorescence and IHC Staining Assay

For immunofluorescence (IF), TG neurons from different groups were fixed in 4% paraformaldehyde in PBS for 20 minutes at room temperature and permeabilized with 0.5% Triton X-100 in PBS for 10 minutes. After washing with PBS twice, the cells were blocked with 2% BSA and 2% FBS in PBS for 1 hour at room temperature. The cells were incubated with the primary antibody of β3-tubulin (1:500, Abcam, ab18207) at 4°C overnight, followed by incubation with FITC-conjugated secondary antibodies (1:500, Abcam, ab150077) and 1 µg/mL of DAPI for 1 hour at room temperature. The slides were examined using fluorescent microscopy at 488 nm.

For IHC, tongue tissue sections were deparaffinized in xylene and rehydrated by incubation in serial ethanol baths. Antigen retrieval was performed through incubation in 10 mmol/L citrate buffer (pH = 6.0) for 20 minutes at 95°C. Endogenous peroxidase activity was inhibited by treatment with 3% H_2_O_2_ for 10 minutes. Then the tissue slides were incubated overnight at 4°C with primary antibodies of β3-tubulin (1:500, Abcam, ab18207), Ki-67 (1:200, Cell Signaling Technology, 12202), caspase-3 (1:500, Cell Signaling Technology, 9662), TGFβ1 (1:500, Abcam, ab215715), PD-L1 (1:200, Cell Signaling Technology, 64988), TNFα (1:100, Sigma, SAB4502982), CD4 (1:200, Abcam ab237722), CD8α (1:100, Cell Signaling Technology, 98941), FoxP3 (1:200, Cell Signaling Technology, 12653), and IFNγ (1:200, Thermo fisher Scientific, PA5-95560). After washing in PBS, the slides were incubated for 2 hours with the secondary antibody (1:500, Abcam, ab6721), and the signal was detected by HRP/DAB Detection Kit (Abcam, ab64261). Quantification was performed by IHC scores in three randomly selected high-power fields (20×) of each slice. The IHC score was calculated by multiplying the positive grade by staining intensity, figuring out an immunoreactivity score of 0–12. The percentage of positive staining was graded as follows: 0 (<1%), 1 (1%–25%), 2 (26%–50%), 3 (51%–75%), or 4 (>75%). Staining intensity was graded as 0 (negative), 1 (weak), 2 (medium), or 3 (strong).

### Statistical Analyses

Standard parametric tests, including Student *t* tests, and one-way or two-way or three-way ANOVAs with Fisher's least significant difference *post hoc* analyses compared the differences between groups using GraphPad Prism 9.10 (RRID: SCR_002798). At a minimum, all experiments were performed in triplicate. Unless otherwise indicated, data are presented as means ± SEM, and significance was accepted at *P* < 0.05.

### Data Availability Statement

The data generated in this study are available within the article and its Supplementary Data.

## Results

### LD Improves the Anti-PD-1 Immunotherapy Efficacy in Mice

**FIGURE 1:**
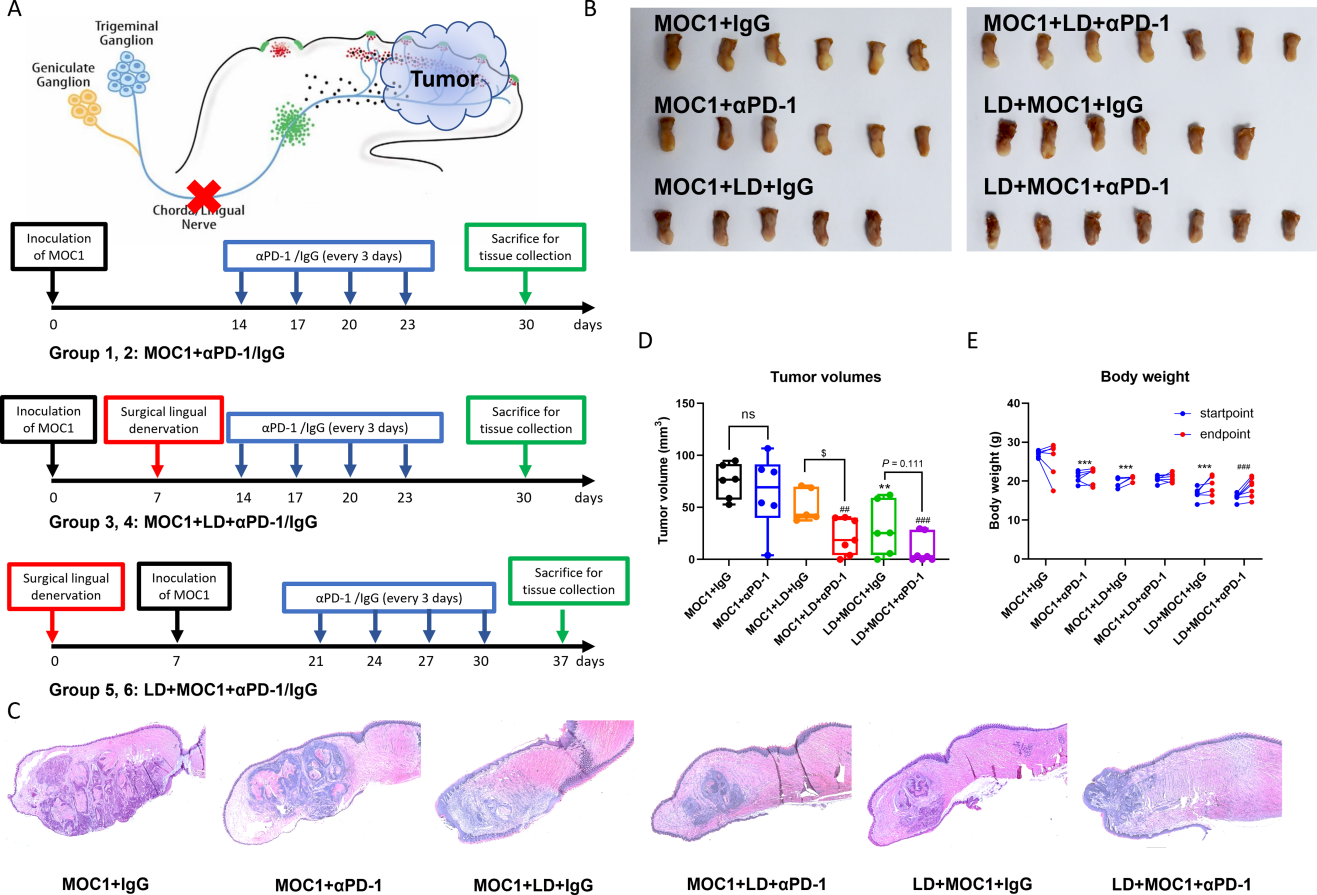
LD inhibits tumor growth and improves anti-PD-1 treatment in OSCC mice. **A,** Experimental procedure of each group. **B,** Tumors harvested in each group (5–7 mice per group). **C,** Tumor volumes of each group at the endpoint (**, *P* < 0.01 vs. MOC1+IgG, respectively; ^##,###^, *P* < 0.01. 0.001 vs. MOC1+αPD-1; ^$^, *P* < 0.05 MOC1+LD+IgG vs. MOC1+LD+αPD-1). **D,** Body weight changes of mice in each group at the endpoint compared with the weight before MOC1 inoculation (***, *P* < 0.001 vs. MOC1+IgG; ^###^, *P* < 0.001 vs. MOC1+αPD-1). **E,** Representative hematoxylin and eosin staining images of tumors in each group.

We investigated the effect of LD on the efficacy of anti-PD-1 immunotherapy in the syngeneic orthotopic OSCC mouse model by inoculating MOC1 cells on the dorsum of tongue. The surgical LD was performed one week before or after cancer cell inoculation, and mice were injected intraperitoneally with αPD-1 or IgG2a 2 weeks after tumor cell inoculation (Fig. 1A). Six groups were set as: MOC1+IgG; MOC1+αPD-1; MOC1+LD+IgG; MOC1+LD+αPD-1; LD+MOC1+IgG; LD+MOC1+αPD-1 (Fig. 1A). As shown in Fig. 1B–D, LD and immunotherapy reduced tumor growth (two-way ANOVA, immunotherapy: *P* = 0.010, LD: *P* < 0.001). In comparison with mice bearing MOC1 tumors treated with isotype IgG2a, treatment with anti-PD-1 therapy shrank the tumor size a little but no significance was reached (*P* = 0.420; Fig. 1C). However, when combined with LD, anti-PD-1 immunotherapy led to a better efficacy by reducing tumor volume than the control treatment (*P* = 0.027 for MOC1+LD +αPD-1 vs. MOC1+LD+IgG and *P* = 0.111 for LD+MOC1+αPD-1 vs. LD+MOC1+IgG; Fig. 1C). Interestingly, in OSCC mice with LD, anti-PD-1 treatment significantly decreased the tumor volume compared with those with no LD (*P* = 0.002 and < 0.001 for MOC1+LD+αPD-1 and LD+MOC1+αPD-1 vs. MOC1+αPD-1, respectively), which illustrated that LD improved the efficacy of anti-PD-1 immunotherapy in OSCC (Fig. 1C). As for the body weight changes, mice with LD had less weight loss at the endpoint when compared with non-LD groups (three-way ANOVA, LD: *P <* 0.001, time × immunotherapy: *P* = 0.338; Fig. 1E). These results demonstrated that LD not only inhibited tumor progression but also improved the efficiency of anti-PD-1 treatment. In converse, neural signaling from the tumor-infiltrating nerve fibers may remodel the tumor microenvironment to an immunosuppressive status which causes resistance to anti-PD-1 immunotherapy. Therefore, we set out to investigate underlying molecular mechanisms.

### Coculturing with Neurons Promotes the Aggressiveness of Tumor Cells

**FIGURE 2:**
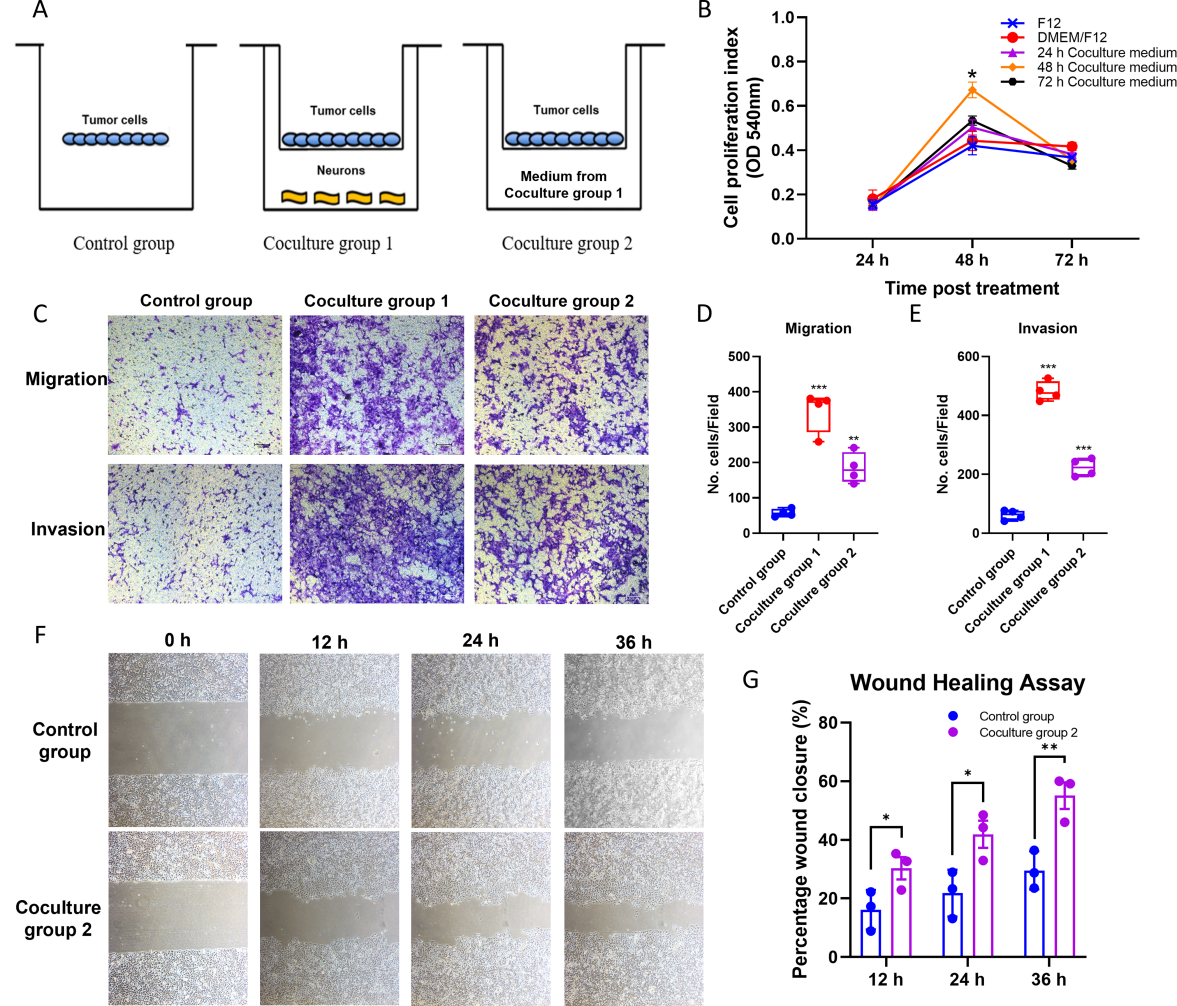
Coculturing with TG neurons promotes proliferation, migration and invasion of OSCC cells. **A,** Neuron-tumor cell coculture models and the control group. **B,** SCC-15 cell viability after treated with the coculture medium of different timepoints (24, 48, and 72 hours) from the coculture group 1 (*, *P* < 0.05 vs. DMEM/F12 group at each timepoint). **C–G,** Migration and invasion activity of SCC-15 cells detected by migration assay, invasion assay, and wound healing assay (quantitative data from three independent experiments are shown in the right, respectively, *^,^**^,^***, *P* < 0.05, 0.01, 0.001).

To investigate the molecular mechanism underlying regulation of tumor activities by the neural signaling, we established the coculture model of TG neurons and oral cancer cells. The coculture group 1 was set with TG sensory neurons in the lower chamber and tumor cells in the upper inserts, while the coculture group 2 was set with the coculture medium from coculture group 1 in the lower chamber and the tumor cells in the upper inserts (Fig. 2A). To identify the optimal coculture time, the viability of tumor cells incubated with the coculture medium at three different timepoints (24, 48, and 72 hours) from coculture group 1 as well as the complete F12 medium or DMEM/F12 medium was investigated. We noticed that SCC-15 cells demonstrated significantly higher proliferative capacity with 48 hours coculture medium after another 48 hours culture (two-way ANOVA, time × group: *P* = 0.019; *post hoc* test, *P* = 0.036; Fig. 2B). Therefore, 48 hours coculture medium from coculture group 1 was chosen to be added into the lower chamber in the coculture group 2 as the attractant, and the 48-hour culture time remained the same for all three groups.

The results of migration and invasion assays revealed that significantly more SCC-15 cells penetrated the membrane of insert in both coculture groups compared with the control group (*P* < 0.001 and *P* = 0.004 for coculture group 1 and 2 vs. control group in migration assay; *P* < 0.001 for coculture group 1 and coculture group 2 vs. control group in invasion assay), suggesting that TG neurons facilitated the migration of SCC-15 cells (Fig. 2C–E). The wound healing assay also showed that the migration ability of SCC-15 cells was significantly higher in coculture group 2 at all timepoints compared with the control group (two-way ANOVA, time × group: *P* < 0.001, *post hoc* test: *P* = 0.035, 0.011 and 0.003 at 12, 24, and 36 hours, respectively; Fig. 2F and G). Similarly, the result of migration and invasion activities in the MOC1 cell line was consistent with that of the SCC-15 cell line (Supplementary Fig. S1). In addition, the IF staining of β3-tubulin showed that TG neurons had more neurites when cocultured with SCC-15 or with 48 hours coculture medium from coculture group 1 compared with the culture in the complete F12 medium (Supplementary Fig. S2), illustrating that SCC-15 cells promoted the neuritogenesis of TG neurons.

### TGFβ-SMAD2 Signaling Upregulates in Tumor Cells in the Neuron-tumor Coculture System

To explore the tumor cell–intrinsic signaling altered after coculturing with TG neurons, we collected the total RNA of SCC-15 cells from the coculture groups and the control group and performed RNA sequencing. The analysis identified 3,058 DEGs in SCC-15 from coculture group 1 compared with the control group, of which 1,583 genes were upregulated and 1,475 genes were downregulated (Supplementary Fig. S2). The analysis also identified 757 DEGs in SCC-15 from coculture group 2, of which 460 genes were upregulated and 297 genes were downregulated (Supplementary Fig. S2).

**FIGURE 3:**
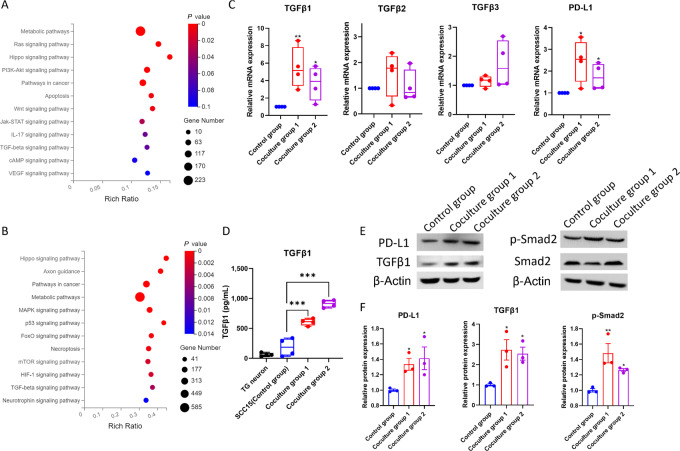
Coculturing with TG neurons activates TGFβ-SMAD2 pathway and upregulates PD-L1 expression of OSCC cells. **A,** KEGG pathway enrichment analysis of the coculture group 1. **B,** KEGG pathway enrichment analysis of the coculture group 2. **C,** Expression levels of TGFβ1, TGFβ2, TGFβ3, and PD-L1 determined by qRT-PCR (*^,^**, *P* < 0.05, 0.01 vs. control group). **D,** The TGFβ1 level in the supernatants of each group determined by ELISA (***, *P* < 0.001 vs. control group). **E** and **F,** Expression levels of TGFβ1, p-SMAD2, SMAD2, and PD-L1 determined by Western blot analysis (*^,^**, *P* < 0.05, 0.01 vs. control group).

To further investigate the pathways that were enriched in each coculture group, we performed gene set enrichment analysis (GSEA) by using the Kyoto Encyclopedia of Genes and Genomes (KEGG) gene sets. The GSEA revealed that TGFβ signaling was enriched in both coculture groups (Fig. 3A and B). There are three isoforms of TGFβ, namely, TGFβ1, TGFβ2, TGFβ3. The mRNA level of *TGFβ1*, *TGFβ2*, *TGFβ3*, and *PD-L1* was detected by qRT-PCR, which showed that only the mRNA level of *TGFβ1* (*P* = 0.009, 0.032 vs. control group; Fig. 3C) and *PD-L1* (*P* = 0.024, 0.047 vs. control group; Fig. 3C) was significantly upregulated in both coculture groups compared with the control group. Our result was consistent with the previous evidence that TGFβ1 shows higher and more widespread upregulation in the tumor microenvironment than the other two isoforms and is more robustly associated with failure of immune checkpoint inhibitor in patients with cancer (24). The TGFβ1 level in the supernatants of coculture groups was sharply increased compared with the control group or TG culture (*P* < 0.001; Fig. 3D). Consistently, protein expression levels of TGFβ1 and PD-L1 were further verified by Western blot analysis, which showed that TGFβ1 (*P* = 0.012, 0.020 vs. control group) together with p-SMAD2 (*P* = 0.004, 0.047 vs. control group) and PD-L1 (*P* = 0.049, 0.023 vs. control group) had significantly higher expression in both coculture groups than the control group (Fig. 3E and F). Interestingly, the expression level of PD-L1 in TG neurons was much higher in the coculture groups compared with the control (*P* < 0.001, *P* = 0.002 vs. TG+F12, respectively; Supplementary Fig. S2), indicating a reciprocal nerve-tumor cross-talk. The result of the MOC1 cell line was also consistent with that in the SCC-15 cell line and showed that both TGFβ-SMAD2 signaling and PD-L1 upregulated in MOC1 cells in the neuron-tumor coculture system (Supplementary Fig. S1).

### Inhibition of TGFβ Signaling by Galunisertib Reversed the Aggressiveness of Tumor Cells in the Neuron-tumor Coculture System

**FIGURE 4:**
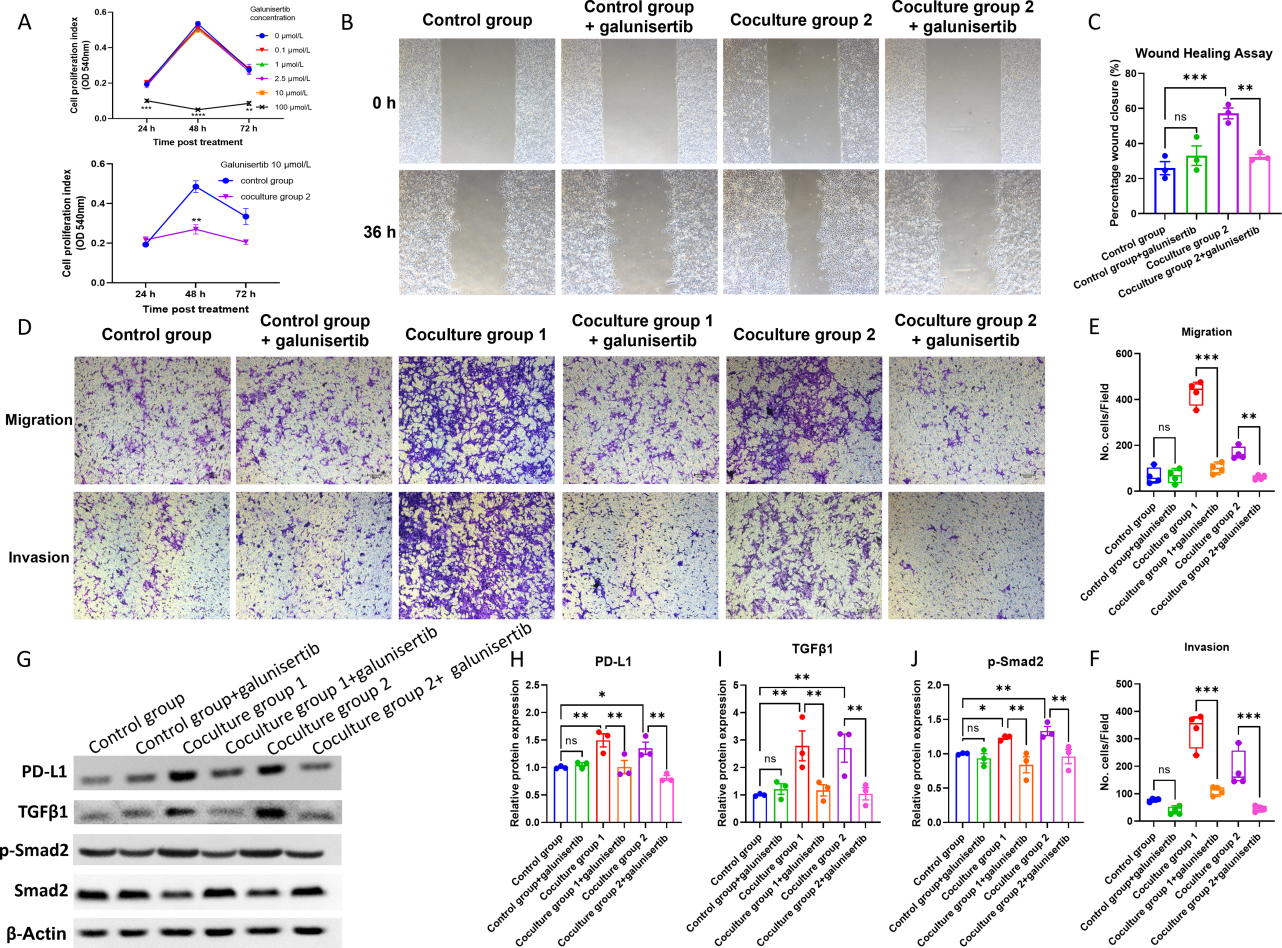
Galunisertib reserves the tumor cell aggressiveness and downregulates TGFβ signaling and PD-L1 expression of tumor cells in the neuron-tumor coculture system. **A,** SCC-15 cell viability after treated with different concertation of galunisertib (**^,^***^,^****, *P* < 0.01, 0.001, 0.0001 vs. 0 µmol/L group) at each timepoint, top; MOC1 cell viability in complete DMEM/F12 (control group) or coculture medium from coculture group 1 (coculture group 2) after treated with 10 µmol/L galunisertib (*, *P* < 0.05, bottom). **B–F,** Migration and invasion activity of SCC-15 cells detected by migration assay, invasion assay, and wound healing assay (quantitative data from three independent experiments are shown in the right, respectively, **^,^***, *P* < 0.01, 0.001). **G–J,** Expression levels of TGFβ1, p-SMAD2, SMAD2, and PD-L1 determined by Western blot analysis (*^,^**, *P* < 0.05, 0.01).

To verify the biological function of the TGFβ-SMAD2 axis in the neuron-tumor coculture system, we took the advantage of a small-molecule inhibitor of TGFβ receptor type I (TGFβRI) kinase galunisertib to inhibit the TGFβ signaling in tumor cells. The treatment of tumor cells in coculture group 2 with galunisertib at concentrations ranging from 0.01 to 10.0 µmol/L exhibited similar proliferative index rates as non-treated tumor cells between 24 and 72 hours (Fig. 4A). At these concentrations, proliferation indices increased from 24 to 48 hours and gradually decreased at 72 hours. Galunisertib at 10 µmol/L decreased the proliferation of SCC-15 cells in coculture group 2 (two-way ANOVA: time × group, *P* < 0.001; *post hoc* test: *P* = 0.002 at 48 hours; Fig. 4A). Therefore, we chose the dose of galunisertib at 10 µmol/L for further experiments because this concentration of drug inhibited tumor cell proliferation in coculture system rather than control group.

To investigate the migration and invasion activities of tumor cells in the neuron-tumor coculture system, the wound healing, migration, and invasion assays was performed. The wound healing assay showed that treatment of galunisertib at 10 µmol/L reversed the high migrating ability of SCC-15 cells in coculture group 2 (*P* = 0.002) but showed no significant effect in SCC-15 culture (Fig. 4B and C). In addition, galunisertib at 10 µmol/L significantly decreased the percentages of tumor cells that migrated (*P* < 0.001 for coculture group 1 vs. coculture group 1+galunisertib and *P* = 0.001 for coculture group 2 vs. coculture group 2+galunisertib) and invaded (*P* < 0.001 for coculture group 1 vs. coculture group 1+galunisertib and for coculture group 2 vs. coculture group 2+galunisertib) in both coculture groups while no obvious effect was observed in the control group (Fig. 4D–F). Moreover, galunisertib at 10 µmol/L reversed the upregulation of TGFβ signaling in SCC-15 cells of both coculture groups (TGFβ1: *P* = 0.005 for coculture group 1 vs. coculture group 1 +galunisertib and for coculture group 2 vs. coculture group 2+galunisertib; p-SMAD2: *P* = 0.003 for coculture group 1 vs. coculture group 1+galunisertib and *P* = 0.005 for coculture group 2 vs. coculture group 2+galunisertib) and PD-L1 (*P* = 0.006 for coculture group 1 vs. coculture group 1+galunisertib and *P* = 0.004 for coculture group 2 vs. coculture group 2+galunisertib) (Fig. 4G–J). The result of the MOC1 cell line was also consistent with that in the SCC-15 cell line and showed that both galunisertib inhibited migration/invasion activities induced by neural coculture and downregulated TGFβ-SMAD2-PD-L1 pathway in tumor cells (Supplementary Fig. S3). Taken together, our results illustrated that inhibition of TGFβ signaling by galunisertib could reverse the tumor aggressiveness in the neuron-tumor cell coculture system.

### LD Decreased Tumor Proliferation and Downregulated TGFβ1 and Enhance CD8^+^ T-cell Activity *In Vivo*

**FIGURE 5:**
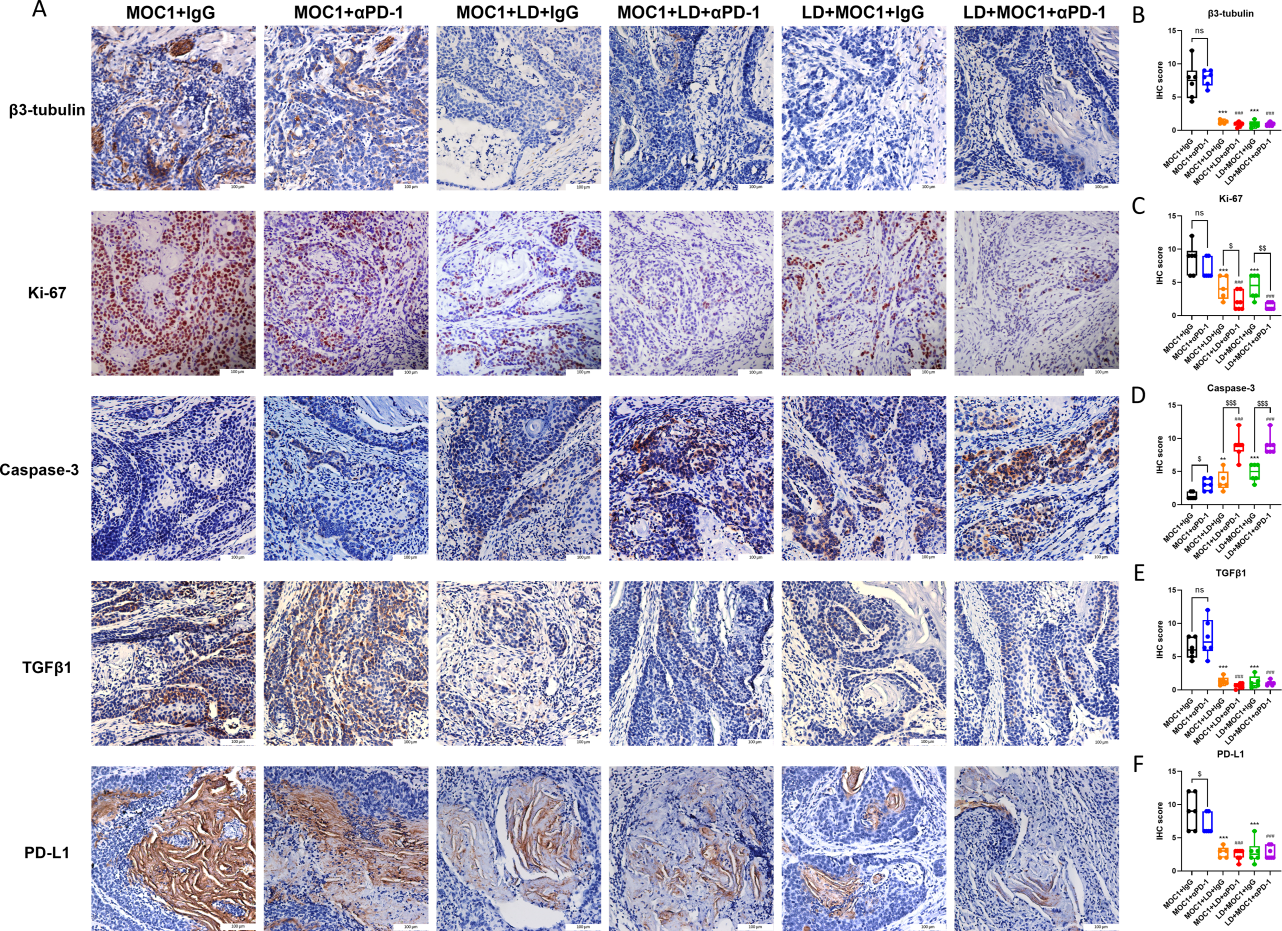
**A,** LD decreased tumor proliferation and downregulated TGFβ1 and PD-L1 expression. **B–F,** The expression levels of β3-tubulin, Ki-67, caspase-3, TGFβ1, and PD-L1 in each group [each bar represents mean ± SD (*n* = 5–7); ***, *P* < 0.001 versus MOC1+IgG; ^###^, *P* < 0.001 vs. MOC1+αPD-1; ^$,^^$$,^^$$$^, *P* < 0.05, 0.01, 0.001 for αPD-1 vs. IgG].

To confirm the surgical LD in the syngeneic orthotopic oral cancer model, the neural fibers were detected by IHC staining of β3-tubulin, which showed that tumors with LD had significantly lower expression of β3-tubulin while mice without LD surgery had higher staining of β3-tubulin within tumor (two-way ANOVA, LD: *P* < 0.001, immunotherapy: *P* = 0.961; Fig. 5A and B). The investigation of tumor proliferation through Ki-67 staining showed that the Ki-67 level in LD tumor was significantly lower compared with non-LD mice with same treatment (two-way ANOVA, LD: *P* < 0.001, immunotherapy: *P* < 0.001), while immunotherapy only decrease tumor proliferation in LD mice rather in non-LD mice (*post hoc* test: *P* = 0.037 for MOC1+LD+αPD-1 vs. MOC1+LD+IgG and *P* = 0.003 for LD+MOC1+αPD-1 vs. LD+MOC1+IgG; Fig. 5A and C), which illustrated that LD inhibited tumor proliferation of OSCC and improved the effect of immunotherapy in reduce tumor growth. In addition, we detected the caspase-3 level to investigate the immune-related tumor cell death in each group. As shown in Fig. 5A and D, LD tumors with immunotherapy has significantly more caspase-3 expression compared with non-LD mice with same treatment or LD mice with IgG treatment (two-way ANOVA, LD: *P* < 0.001, immunotherapy: *P* < 0.001).

To further verify the role of TGFβ signaling in tumor progression induced by neural involvement, the expression of TGFβ1 in the syngeneic orthotopic oral cancer model was detected by IHC staining. OSCC mice without LD has similar TGFβ1 expression level but much higher than those undertaking surgical LD (two-way ANOVA, LD: *P* < 0.001, immunotherapy: *P* = 0.644), which was consistent with the cellular assay that neural involvement enhanced TGFβ signaling in tumor (Fig. 5A and E). Moreover, anti-PD-1 treatment significantly decreased PD-L1 level in non-LD tumor but not in LD tumor (two-way ANOVA, LD: *P* < 0.001, immunotherapy: *P* = 0.116), while LD tumors had much lower PD-L1 level compared with non-LD tumors with same treatment (*P* < 0.001; Fig. 5A and F). These results illustrated that LD slowed down tumor growth and reduced tumor aggressiveness prior to immunotherapy.

**FIGURE 6:**
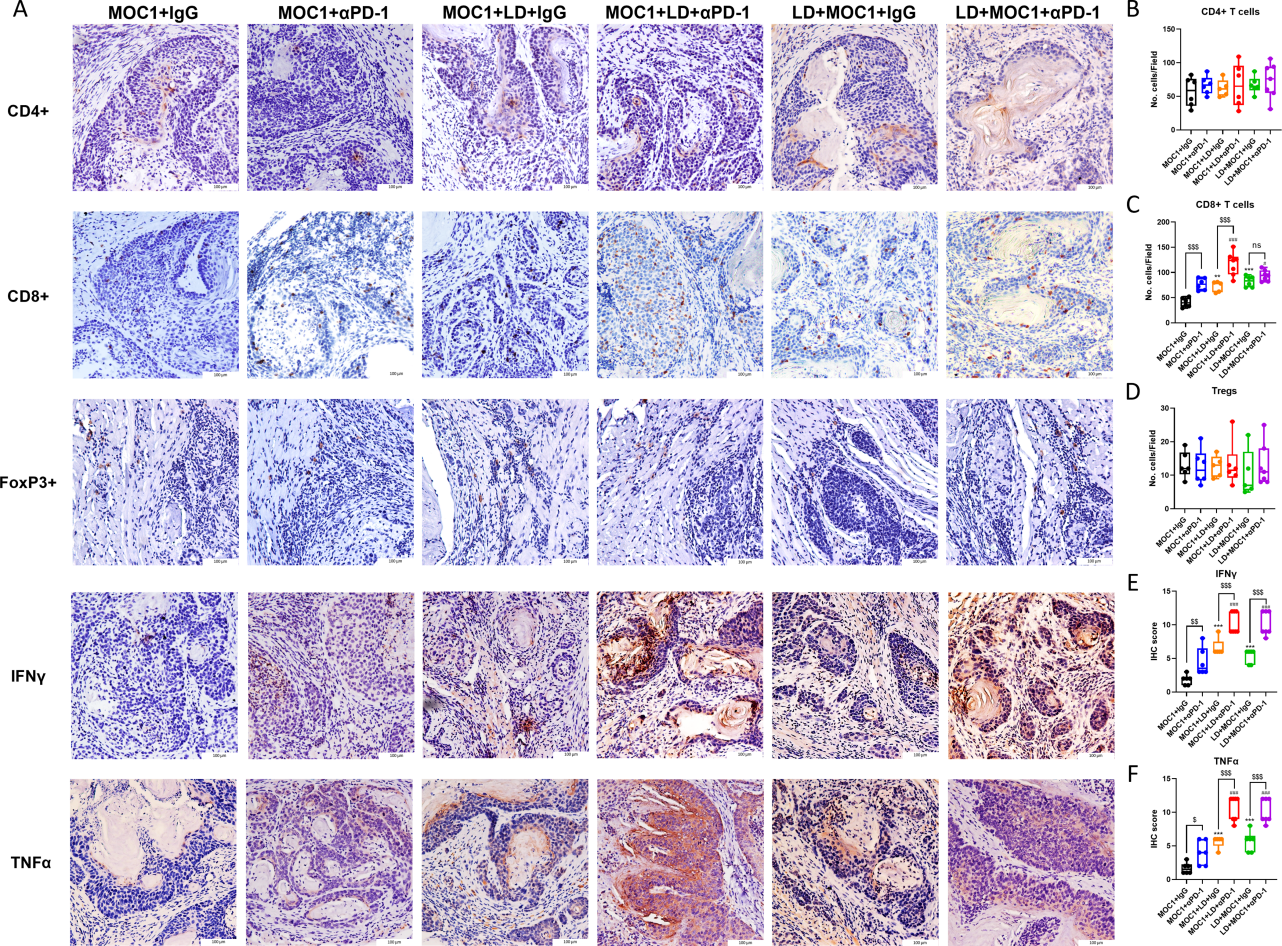
**A,** LD enhances CD8^+^ T-cell activities. **B–F,** The expression levels of CD4^+^, CD8^+^ T cells, Tregs, IFNγ, and TNFα in each group [each bar represents mean ± SD (*n* = 5–7); **^,^***, *P* < 0.01, 0.001 vs. MOC1+IgG; ^#^,^###^, *P* < 0.05, 0.001 vs. MOC1+αPD-1; ^$^^,^^$$$^, *P* < 0.05, 0.001 αPD-1 vs. IgG].

To evaluate T-cell responses triggered by neural infiltration, we performed IHC staining and assessed the infiltration of CD4^+^, CD8^+^ T cells, and regulatory T cells (Treg) into tumors and quantified IFNγ and TNFα expression levels. We demonstrated no significant differences in the infiltration of either CD4^+^ T cells or Tregs of each group (two-way ANOVA, LD × immunotherapy: *P* = 0.999 and 0.552, respectively; Fig. 6A, B, and D). However, LD tumors had more abundant CD8^+^ T cells compared with non-LD groups with same treatment, and immunotherapy also increased CD8^+^ T-cell infiltration in both LD tumors and non-LD tumors (two-way ANOVA, LD: *P* < 0.001, immunotherapy: *P* < 0.001; Fig. 6A and C). In addition, the expression level of IFNγ and TNFα was much higher in tumors with LD or anti-PD-1 treatment (two-way ANOVA, LD: *P* < 0.001, immunotherapy: *P* < 0.001; Fig. 6A, E, and F). Also, sham LD surgery did not change the tongue immune microenvironment because sham LD tongue had no significant difference of CD4^+^, CD8^+^ T-cell, and Treg infiltration compared with naïve tongue (Supplementary Fig. S4). However, the infiltration of CD8^+^ T cells was detected only in denervated tongues rather than sham-LD or naïve tongues (Supplementary Fig. S4). Mice with sham LD surgery before or after tumor cell inoculation had the similar tumor microenvironment at each timepoint (days 10, 20, and 30 after tumor cell inoculation) compared with control mice without surgery (Supplementary Fig. S5). These results illustrated that LD enhanced the infiltration of CD8^+^ T cells into tumors and promoted IFNγ and TNFα levels within tumor, which was essential for improving the efficacy of immunotherapy in mice bearing OSCC tumors.

## Discussion

Tumor innervation and neural invasion within the tumor microenvironment have been investigated extensively in recent decades and may have the potential to explain several key puzzles in cancer, such as tumor progression and immune evasion (25). We appreciate all clinical evidence on the correlation of high nerve density with poor prognosis in OSCC, which inspired our research on nerve-cancer cross-talk (5, 6). In the current study, we established a neuron-tumor cell coculture system and found that tumor cells were more aggressive when coculturing with sensory neurons from TGs, which may attribute to the activation of TGFβ signaling, followed by the upregulation of PD-L1 expression in tumor cells. This process could be attenuated by a specific inhibitor of TGFβ signaling, galunisertib. In addition, LD in the syngeneic orthotopic oral cancer model not only suppressed tumor growth, decreased tumor aggressiveness but also created an immune-active microenvironment by downregulation of TGFβ signaling and enhanced CD8^+^ T-cell activity, leading to the improved anti-PD-1 treatment. Our study reveals the essential role of neural involvement in tumor progression and immunotherapy resistance, and the data presented herein will inspire further research on adjuvant therapy targeting the nervous system in cancer therapy.

### Nerve-mediated Tumor Aggressiveness

As a type of neurotrophic tumor, 80% of OSCC cases have been detected with neural involvement (26). Intratumoral neural infiltration and tumor innervation correlating to tumor progression and poor clinical outcomes has been confirmed in many solid types of cancer including OSCC (27). The current study established the neuron-tumor cell coculture model and mimicked the tumor aggressive behaviors mediated by neural involvement *in vitro*, and also investigated the inhibition effect of surgical denervation on tumor growth in immunocompetent OSCC mice. Combining the results from both *in vitro* and *in vivo* models, we highlighted the essential role of nerve–cancer interaction in tumor development.

Lingual nerves project from TG, innervate the tongue and supply sensation of touch, pain, temperature and taste for the tongue (28). Hence, we performed lingual nerve neurectomy to create denervation *in vivo*, and chose TG sensory neurons to coculture with tumor cells *in vitro* to mimic intratumoral neural infiltration. Whether denervation process performed before or after tumor cell inoculation seems to be no different in its impact on attenuating tumor growth or improving immunotherapy in our study, which illustrated that innervation not only facilitates tumor growth but also tumorigenesis. Previous studies used dorsal root ganglion coculturing with cancer cells to investigate both the neurotropic effect of tumor cells and the neurite outgrowth process (29). However, the dorsal root ganglion and TG sensory neurons are different from each other, including but not limited to, the different distribution of neuron types and nociceptors (30, 31). Thus, our *in vitro* coculture system is more approximate to the nerve-tumor cross-talk in OSCC.

Nerve fibers were recognized in cancer decades ago (32), and the studies since revealed that perineural invasion (PNI), the passive invasion of locoregional nerves to tumors, led to tumor proliferation and metastasis and was associated with poor prognosis (33). Later, tumor innervation was identified as an active recruitment process of nerves into the tumor microenvironment and contributed to tumorigenesis and tumor growth (9), providing new perspectives to nerve–tumor interactions. Therefore, the interaction between nerve and cancer is reciprocal. Our results illustrated that tumor cells facilitated neuritogenesis of TG neurons, which was consistent with the previous study showing that extracellular vesicles from OSCC cell culture enhanced neurite outgrowth of murine dorsal root ganglion (8). This phenomenon may attribute to the tumor cells secreting some neurotrophic factors (34) or axon guidance regulators (35) to the microenvironment, leading to a stimulation of neurogenesis.

### Nerve-mediated Immunosuppression in the Tumor Microenvironment

In the tumor microenvironment, infiltrated nerves exert an inhibition role on anticancer immunity in at least three different ways. First, some tumor-infiltrating nerves express a high level of PD-L1, which was found in prostate cancer and a higher density of PD-L1 on tumor-associated nerves was associated with a worse prognosis (36). And our results also showed a much higher expression level of PD-L1 on TG neuron cocultured with tumor cells. Second, neural signaling or neuroactive molecules secreted by intratumoral nerve fibers can directly interact with immune cells within the tumor microenvironment, because immune cells express receptors for plenty of neurotransmitters and neuropeptides. For example, β-adrenergic activation induced macrophage differentiation to the immunosuppressive M2 phenotype in the breast cancer mouse model (37). Calcitonin gene-related peptide (CGRP) from sensory neurons promoted the exhaustion of cytotoxic CD8^+^ T cells in melanoma and HNSCC (38, 39). Third, neural involvement affects the cancer-immunity cycle through dysregulating tumor cell–intrinsic signaling pathways (27). For example, nerve-derived glial cell line–derived neurotrophic factor (GDNF) activated JAK2-STAT1 signaling to further enhance PD-L1 expression in head and neck cancer (40).

Our results conformed to the second and third ways mentioned above. The presented data demonstrated that nerve-cancer cross-talk not only activated TGFβ signaling and upregulated PD-L1 on tumor cells, but also decreased IFNγ and TNFα expression levels and reduced CD8^+^ T-cell activity in tumor parenchyma, resulting in impaired anticancer immunity and decreased immune checkpoint blockade responses. Denervation of tumor may rescue antitumor immunity by inhibition of tumor cell–derived TGFβ signaling or blockade direct interaction between neuroactive molecules and immune cells within tumor microenvironment. Our study did not focus on specific type of neural signaling such as β-adrenergic and CGRP signaling as previous studies (37–39), but transected lingual nerve to tongue, and it might be a better method for stopping all neural signaling from sensory nerves. Our study extended previous findings in nerve-cancer cross-talk and cancer–immune interaction. Mounting evidence has been focused on the role of neural signaling on tumor-intrinsic pathways (9) and the effect of tumor-intrinsic pathways in antitumor immunity (41), while our study connected these two parts.

Tumors with higher PD-L1 expression tend to have better response rates to anti-PD-1 treatments in many types of cancer, and current evidence in HNSCC suggests that combined positive score (the sum total of PD-L1–positive tumor cells, lymphocytes, and macrophages divided by the total number of tumor cells) acts as an effective evaluation to screen patients for immunotherapy (10). It seems to be contradictory with our results that non-LD tumor has higher PD-L1 expression level but worse response to immunotherapy. Actually, the relationship between PD-L1 expression in tumor microenvironment and the response to immunotherapy is complicated and controversial. Clinically, some patients with PD-L1–negative HNSCC could still benefit from immunotherapy (42), while higher expression level of PD-L1 seems to be not correlated with better survival in longer-term follow-up of immunotherapy (43). PD-L1 expression is significantly higher on tumor exosomes than on tumor cells and knockout of PD-L1 on tumor exosomes promote antitumor immunity and inhibit tumor growth (44). According to Fig. 5A, PD-L1 are most expressed in tumor stroma rather than tumor cell membranes, so a possible explanation for our results is that non-LD groups have much more expression of PD-L1 in tumor exosomes and distribute in tumor stroma, which could antagonize immunotherapy by binding to the antibody itself and induce immune resistance.

### Immunosuppressive Role of TGFβ Signaling in the Tumor Microenvironment

In our study, TG neurons upregulated PD-L1 expression via activating TGFβ-SMAD2 signaling and this process was attenuated by TGFβ signaling inhibitor galunisertib *in vitro*, and LD decreased both TGFβ1 and PD-L1 expression and enhance CD8^+^ T-cell activity *in vivo*. However, our study did not provide specific mechanisms of TGFβ signaling upregulating PD-L1 and impairing CD8^+^ T-cell activity within the tumor microenvironment. In fact, the immunosuppressive role of TGFβ signaling in cancer has been extensively explored. In a metastatic urothelial cancer cohort, patients’ response to anti-PD-L1 immunotherapy was positively correlated to CD8^+^ T-effector cell infiltration while negatively correlated to TGFβ signaling in cancer-related fibroblasts (19). Selective inhibition of TGFβ1 activation combined with anti-PD-1 treatment increased intratumoral CD8^+^ T cells and decreased immunosuppressive myeloid cells, resulting in profound antitumor responses (24). Dual inhibition of TGFβ1 and PD-L1 combined with radiotherapy remodeled the tumor microenvironment via increasing tumor-infiltrating leukocytes (45). Our results together with previous studies emphasized the immunosuppressive role of TGFβ signaling in cancer.

### Targeting Nerve-cancer Cross-talk as an Adjuvant Therapeutic Approach for Cancer

The data presented herein together with previous studies on the cancer-neuro-immune network provide a rationale for targeting intratumoral neural signaling as an adjuvant therapeutic approach for clinical cancer management. The following several strategies can be used as the intervention of tumor innervation or perineural invasion, some of them have already been performed in preclinical studies or even clinical trials.

First, direct blockade neuronal input by surgical or chemical denervation is an obvious and efficient approach for solid tumors which have specific nerve supply and display nerve-mediated aggressiveness. The feasibility and validity of denervation have been proved in several types of cancer in rodent models, including pancreatic ductal adenocarcinoma (46), fibrosarcoma (47), prostate cancer (48), gastric cancer (49), and melanoma (50). Our study together with a previous study also showed that LD slowed down OSCC growth in the mouse model (8). A clinical study with 37 patients with gastric cancer found that denervation of the stomach decreased the recurrence rate after distal gastrectomy (49).

Second, blockade or inhibition of neural signaling from intratumoral nerves is a more broadly applicable strategy. Both preclinical and clinical studies have revealed that β-blockers (inhibitors of β-adrenergic signaling) modulate cancer progression, improve immunotherapy, and prolong survival in several types of cancer (8, 51, 52). NGF or TrkA blockade decreases tumor proliferation, invasion, metastasis, and PNI in oral cancer mice (53, 54). Fortunately, anti-neurotrophic drugs, including Trk receptor inhibitors (entrectinib, larotrectinib; ref. 55), anti-NGF mAb (tanezumab; ref. 56), and CGRP inhibitors (eptinezumab, erenumab; ref. 57) have been approved by FDA and are potential to be repurposed as adjuvant therapies to regulate the intratumoral neural component.

Third, targeting tumor cell–intrinsic pathways in response to neural signaling or neuropeptides holds the potential for most broad applicability across almost all tumors, even including those only affected by neural signaling but without tumor innervation. Previous studies have demonstrated some tumor cell–intrinsic pathways alteration in nerve-cancer cross-talk, such as Hippo signaling activated by vagal nerve innervation in gastric cancer (58), MAPK and PI3K-AKT signaling enhanced by vagal nerve innervation in pancreatic ductal adenocarcinoma (59), JAK2-STAT1 signaling upregulated by nerve-derived GDNF in head and neck cancer (40) and so on. As shown in our study, TGFβ signaling upregulated by TG neurons in OSCC is also a target for invention of nerve-cancer cross-talk.

### Limitations

There were several limitations of this study. First, though the role of nerve-cancer cross-talk in tumor progression and immunotherapy in oral cancer is validated in both cellular and animal research, the clinical patient-derived samples are not included in this study. Therefore, the clinical impact awaits further validation in prospective or retrospective research. Second, the animal model in this study is a syngeneic orthotopic tongue squamous cell carcinoma model, and other OSCC models such as 4-nitroquinoline-1-oxide (4-NQO)-induced OSCC model are needed to validate the findings of this study, because 4-NQO–induced OSCC model mimics the stepwise progression of oral cancer and the nerve–cancer interaction can be investigated across each stage of carcinogenesis. Third, we focused on the comprehensive effect of intratumoral nerve on oral cancer. However, the specific neural signaling involved in this process needs further investigation, which may provide potential targets for adjuvant therapy research.

### Conclusion

Our study demonstrated that denervation of OSCC inhibited tumor growth and improved anti-PD-1 immunotherapy through downregulating TGFβ signaling and PD-L1 expression and activating CD8^+^ T-cell activities within the tumor microenvironment. We also developed a neuron-tumor cell coculture system *in vitro* and illustrated that nerve-cancer cross-talk promoted aggressiveness through upregulation of TGFβ signaling and PD-L1 expression in tumor cells. The data presented herein not only revealed possible mechanisms for nerve-derived cancer progression and impaired antitumor immunity, but also pave the way for new strategy research on potential adjuvant therapeutic approach targeting nerve-cancer cross-talk in OSCC. The interactions between nerves and multiple types of cells in the tumor microenvironment are intricate, and thus there is still a long way to unravel the overall perspective of the cancer-neuron-immune network and discover novel anti-neurogenic targets for treating cancer.

## Supplementary Material

Supplementary Figure 1 Supplementary Figure 1. Coculturing with TG neurons promotes aggressiveness of MOC1 cells and activates TGFbeta signaling.

Supplementary Figure 2 Supplementary Figure 2. (A) Representative immunofluorescence of neuritogenesis in different coculture groups. (B) Heatmaps displaying DEGs between coculture groups and the control group. (C) Volcano maps displaying DEGs between coculture groups and the control group. (D) The expression levels of PD-L1 in different coculture groups and control group.

Supplementary Figure 3 Supplementary Figure 3. Galunisertib reserves the tumor cell aggressiveness and downregulates TGFbeta signaling and PD-L1 expression of tumor cells in the neuron-tumor coculture system.

Supplementary Figure 4 Supplementary Figure 4. CD4+, CD8+ T cells and Tregs expression in naïve tongues, tongues 10, 20 and 30 days post sham lingual denervation surgery and tongues 7 days post lingual denervation surgery in mice.

Supplementary Figure 5 Supplementary Figure 5. CD4+, CD8+ T cells and Tregs expression in mice with sham lingual denervation surgery before or after MOC1 tumor cell inoculation or without surgery.
