# Supplementary material for: Lingual Denervation Improves the Efficacy of Anti-PD-1 Immunotherapy in Oral Squamous Cell Carcinomas by Downregulating TGFβ Signaling
Source: Cancer Res Commun. 2024 Feb 15;4(2):418–30. doi: 10.1158/2767-9764.CRC-23-0192 (PMC10868515; doi:10.1158/2767-9764.CRC-23-0192)
Supplement: Supplementary Figure 1 — Coculturing with TG neurons promotes aggressiveness of MOC1 cells and activates TGFbeta signaling. [file crc-23-0192-s01.pdf]

Supplementary Figure 1

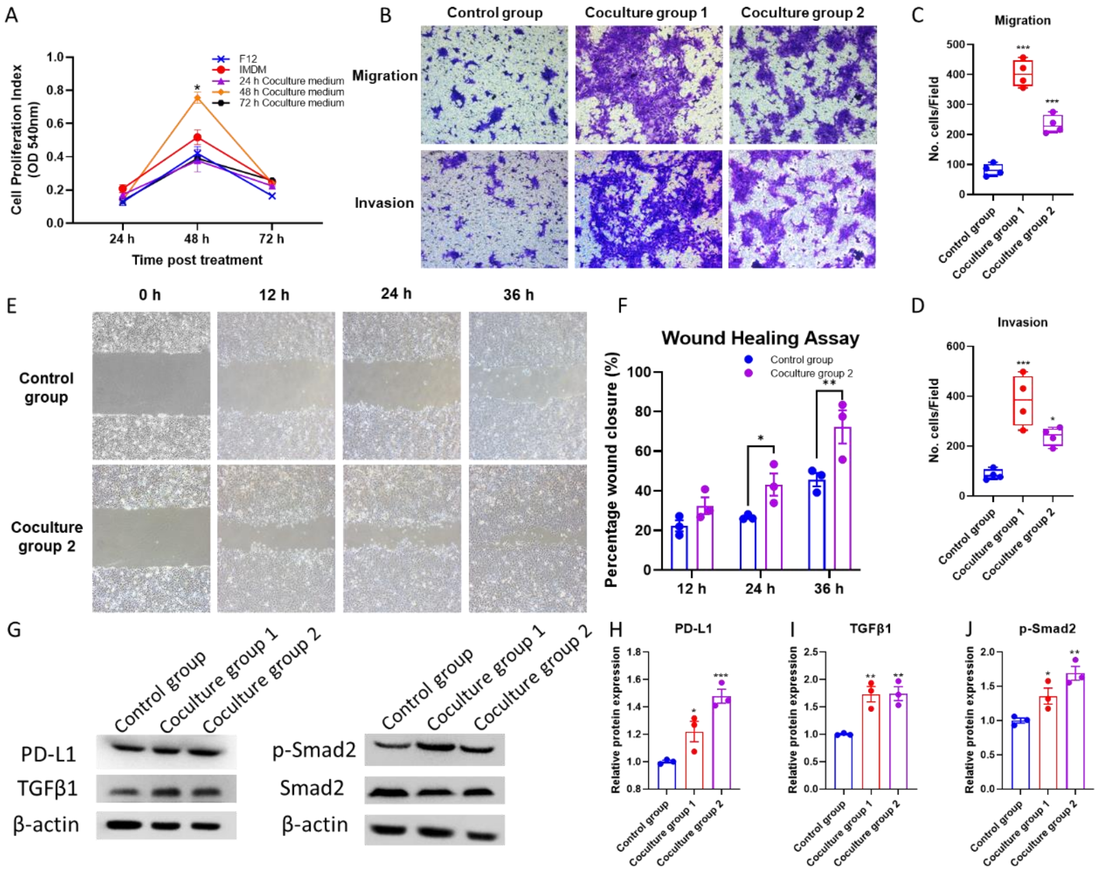

Supplementary Figure 1: Coculturing with TG neurons promotes aggressiveness of MOC1 cells and activates TGFβ signaling. (A) MOC1 cell viability after treated with the coculture medium of different time points (24 h, 48 h and 72 h) from the coculture group 1 (*$p < 0.05$ vs. IMDM group at each time point). (B-F) Migration and invasion activity of MOC1 cells detected by migration assay, invasion assay and wound healing assay (*, **, *** $p < 0.05, 0.01, 0.001$ vs. control group). (G) Expression levels of TGFβ1, p-Smad2, Smad2 and PD-L1 determined by Western blot (*, **, *** $p < 0.05, 0.01, 0.001$ vs. control group).
