# Supplementary material for: Lingual Denervation Improves the Efficacy of Anti-PD-1 Immunotherapy in Oral Squamous Cell Carcinomas by Downregulating TGFβ Signaling
Source: Cancer Res Commun. 2024 Feb 15;4(2):418–30. doi: 10.1158/2767-9764.CRC-23-0192 (PMC10868515; doi:10.1158/2767-9764.CRC-23-0192)
Supplement: Supplementary Figure 2 — (A) Representative immunofluorescence of neuritogenesis in different coculture groups. (B) Heatmaps displaying DEGs between coculture groups and the control group. (C) Volcano maps displaying DEGs between coculture groups and the control group. (D) The expression levels of PD-L1 in different coculture groups and control group. [file crc-23-0192-s02.pdf]

Supplementary Figure 2

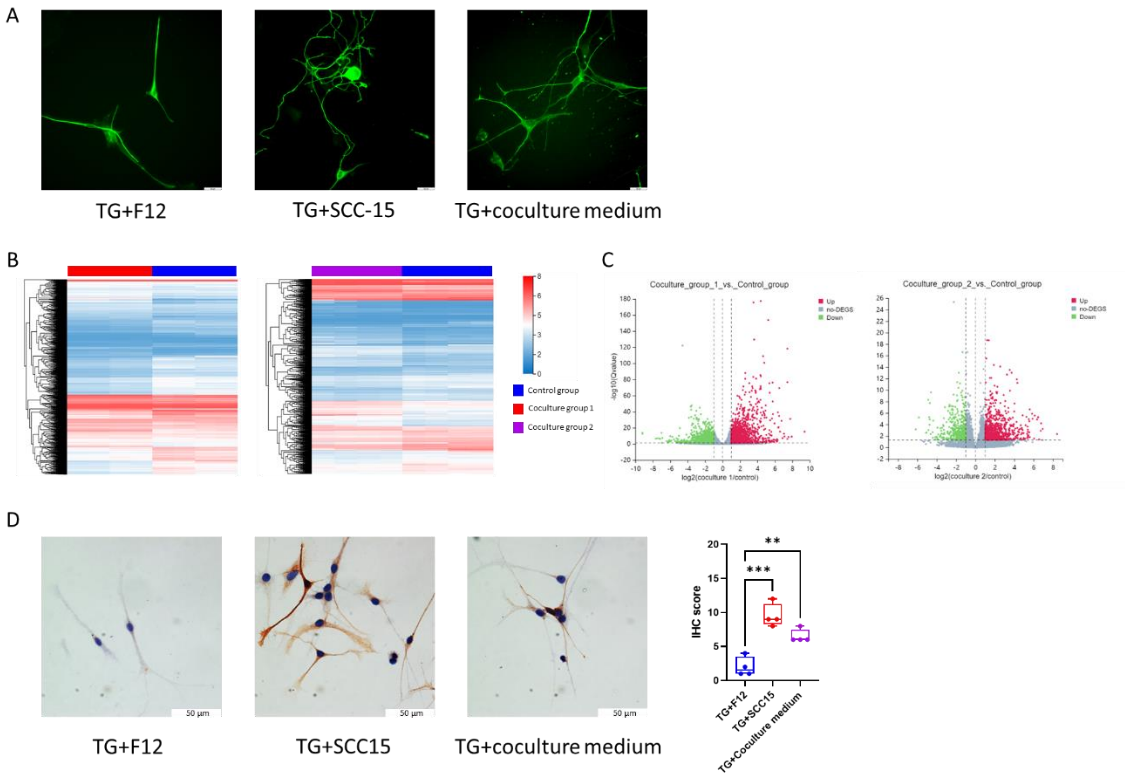

Supplementary Figure 2: (A) Representative immunofluorescence of neuritogenesis in different coculture groups. (B) Heatmaps displaying DEGs between coculture groups and the control group. (C) Volcano maps displaying DEGs between coculture groups and the control group. (D) The expression levels of PD-L1 in different coculture groups and control group (**, *** $p < 0.01$, 0.001 vs. TG+F12 group).
