# Supplementary material for: Lingual Denervation Improves the Efficacy of Anti-PD-1 Immunotherapy in Oral Squamous Cell Carcinomas by Downregulating TGFβ Signaling
Source: Cancer Res Commun. 2024 Feb 15;4(2):418–30. doi: 10.1158/2767-9764.CRC-23-0192 (PMC10868515; doi:10.1158/2767-9764.CRC-23-0192)
Supplement: Supplementary Figure 3 — Galunisertib reserves the tumor cell aggressiveness and downregulates TGFbeta signaling and PD-L1 expression of tumor cells in the neuron-tumor coculture system. [file crc-23-0192-s03.pdf]

Supplementary Figure 3

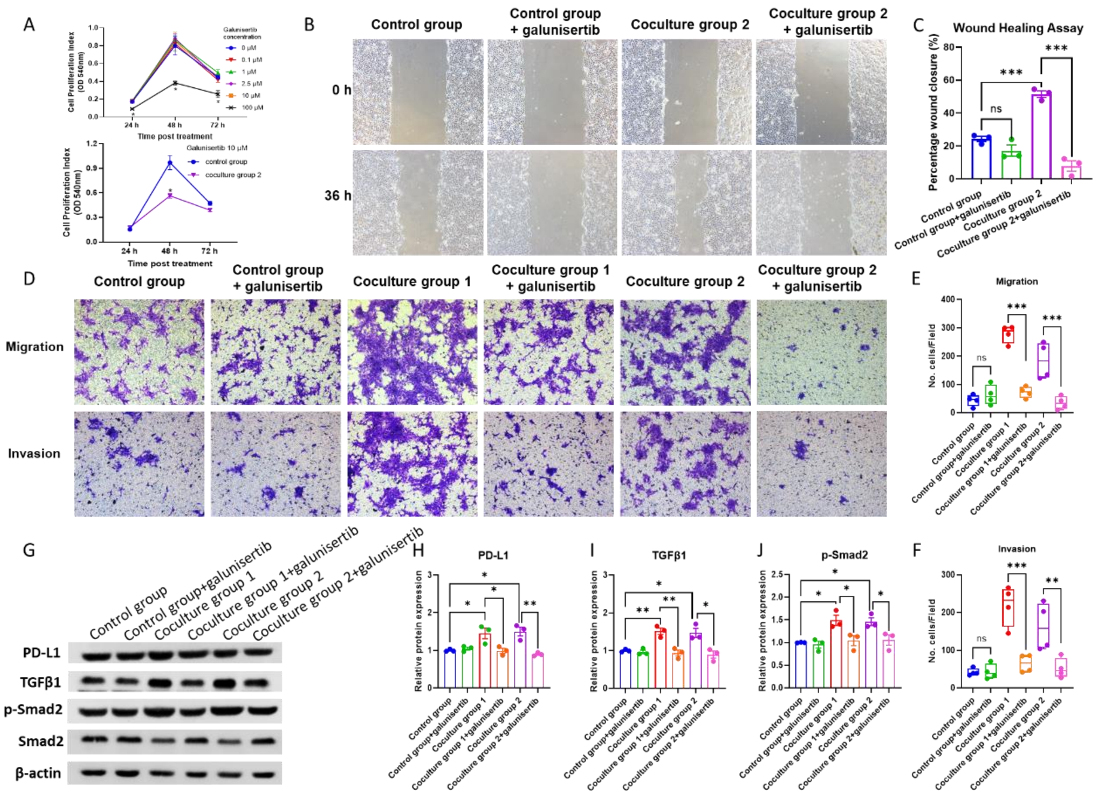

Supplementary Figure 3: Galunisertib reserves the tumor cell aggressiveness and downregulates TGFβ signaling and PD-L1 expression of tumor cells in the neuron-tumor coculture system. (A) MOC1 cell viability after treated with different concertation of galunisertib (**p* < 0.05 vs. 0 μM group at each time point; upper panel); MOC1 cell viability in complete IMDM MOC line media (control group) or coculture medium from coculture group 1 (coculture group 2) after treated with 10 μM galunisertib (**p* < 0.05, lower panel). (B-F) Migration and invasion activity of MOC1 cells detected by migration assay, invasion assay and wound healing assay (**, *** *p* < 0.01, 0.001). (G-J) Expression levels of TGFβ1, p-Smad2, Smad2 and PD-L1 in MOC1 cells determined by Western blot (*, ** *p* < 0.05, 0.01).
