# Supplementary material for: Lingual Denervation Improves the Efficacy of Anti-PD-1 Immunotherapy in Oral Squamous Cell Carcinomas by Downregulating TGFβ Signaling
Source: Cancer Res Commun. 2024 Feb 15;4(2):418–30. doi: 10.1158/2767-9764.CRC-23-0192 (PMC10868515; doi:10.1158/2767-9764.CRC-23-0192)
Supplement: Supplementary Figure 4 — CD4+, CD8+ T cells and Tregs expression in naïve tongues, tongues 10, 20 and 30 days post sham lingual denervation surgery and tongues 7 days post lingual denervation surgery in mice. [file crc-23-0192-s04.pdf]

Supplementary Figure 4

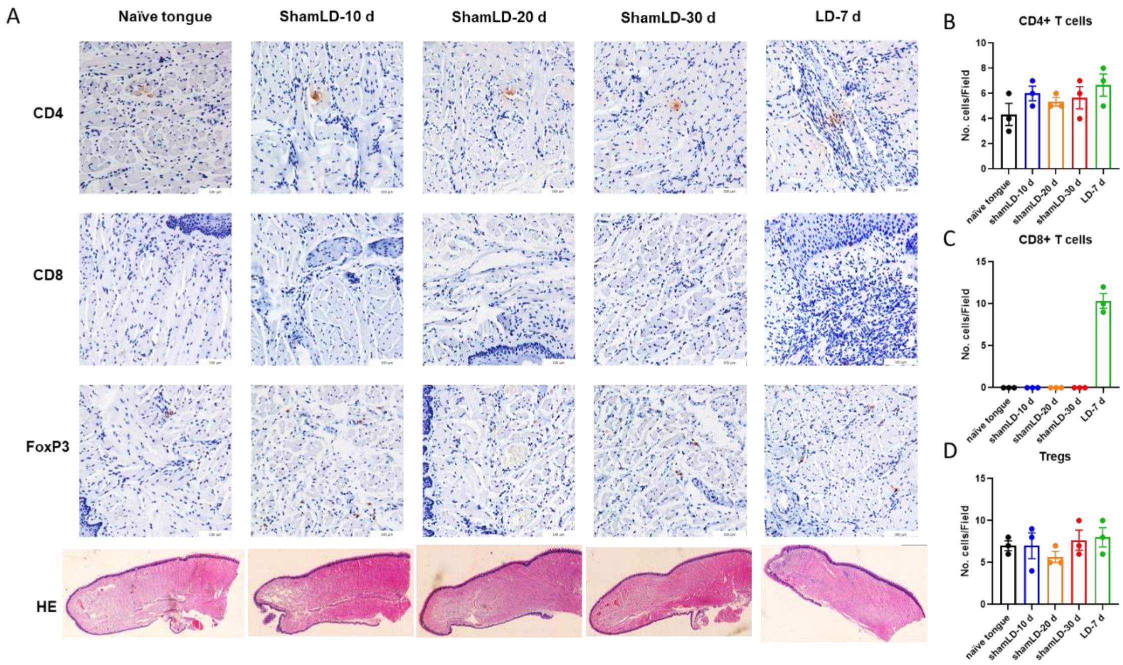

Supplementary Figure 4: CD4+, CD8+ T cells and Tregs expression in naïve tongues, tongues 10, 20 and 30 days post sham lingual denervation surgery and tongues 7 days post lingual denervation surgery in mice (n =3 per group).
