# Supplementary material for: Lingual Denervation Improves the Efficacy of Anti-PD-1 Immunotherapy in Oral Squamous Cell Carcinomas by Downregulating TGFβ Signaling
Source: Cancer Res Commun. 2024 Feb 15;4(2):418–30. doi: 10.1158/2767-9764.CRC-23-0192 (PMC10868515; doi:10.1158/2767-9764.CRC-23-0192)
Supplement: Supplementary Figure 5 — CD4+, CD8+ T cells and Tregs expression in mice with sham lingual denervation surgery before or after MOC1 tumor cell inoculation or without surgery. [file crc-23-0192-s05.pdf]

Supplementary Figure 5

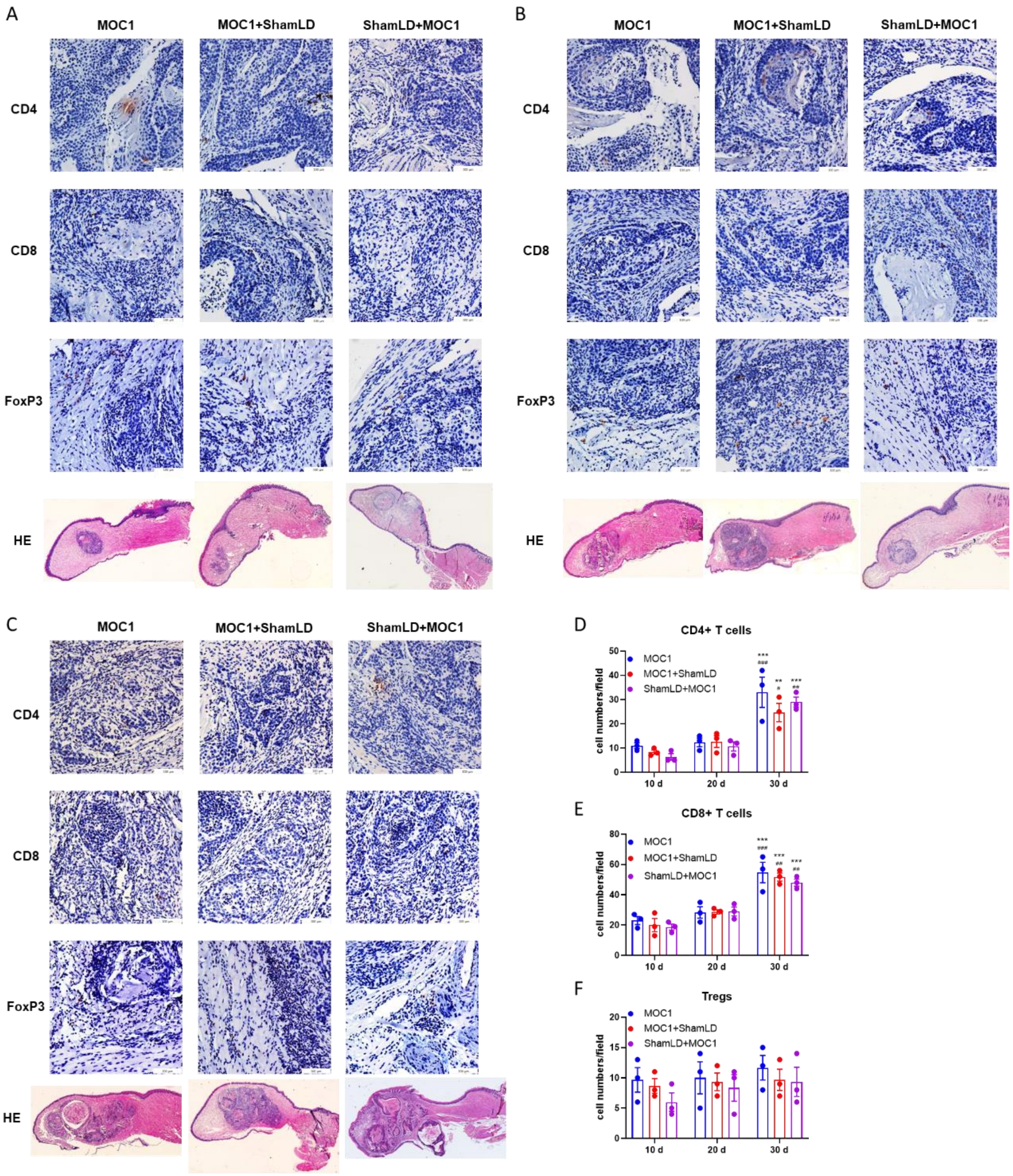

Supplementary Figure 5: CD4+, CD8+ T cells and Tregs expression in mice with sham lingual denervation surgery before or after MOC1 tumor cell inoculation or without surgery. (A)10 days after MOC1 inoculation. (B) 20 days after MOC1 inoculation. (C) 30 days after MOC1 inoculation. (D, E, F) statistical analysis of CD4+, CD8+ T cells and Tregs expression in each group (n =3 per group, **, *** $p < 0.01$, 0.001 vs the same group at 10-day; $^{\#}$, $^{\#\#}$, $^{\#\#\#}$ $p < 0.05$, 0.01, 0.001 vs the same group at 20-day).
